# Approaches to Deprescribing Proton Pump Inhibitors in Clinical Practice: A Systematic Review

**DOI:** 10.3390/jcm13206283

**Published:** 2024-10-21

**Authors:** Andrea Rossi, Lara Perrella, Stefano Scotti, Elena Olmastroni, Federica Galimberti, Ilaria Ardoino, Valentina Orlando, Enrica Menditto, Carlotta Franchi, Manuela Casula

**Affiliations:** 1 Epidemiology and Preventive Pharmacology Service (SEFAP), Department of Pharmacological and Biomolecular Sciences (DiSFeB), University of Milan, 20133 Milan, Italy; andrea.rossi1@unimi.it (A.R.); elena.olmastroni@unimi.it (E.O.); 2 IRCCS MultiMedica, 20099 Sesto San Giovanni, Italy; stefano.scotti@multimedica.it (S.S.); federica.galimberti@multimedica.it (F.G.); 3 Center of Pharmacoeconomics and Drug Utilization Research (CIRFF), Department of Pharmacy, University of Naples Federico II, 80138 Naples, Italy; lara.perrella@unina.it (L.P.); valentina.orlando@unina.it (V.O.); enrica.menditto@unina.it (E.M.); 4 Laboratory of Pharmacoepidemiology and Human Nutrition, Department of Health Policy, Istituto di Ricerche Farmacologiche Mario Negri IRCCS, 20156 Milan, Italy; ilaria.ardoino@marionegri.it (I.A.); carlotta.franchi@marionegri.it (C.F.)

**Keywords:** proton pump inhibitors (PPIs), deprescribing, intervention, symptoms

## Abstract

**Background**: Proton pump inhibitors (PPIs) are some of the most frequently prescribed medications, but they are often used inappropriately, either being prescribed without a clear indication or continued for longer than necessary. In such cases, deprescribing is recommended. However, despite its proven effectiveness, the implementation of deprescribing in clinical practice remains inconsistent and varied, making it challenging to identify the most effective strategies. The goal is to provide a comprehensive outline of deprescribing interventions for PPI therapy implemented across various settings and by different healthcare professionals. **Methods**: The study is designed to be a systematic review of the published literature. PubMed, Embase, and Web of Science databases were searched from 1 January 1989 (the first PPI on the market) to 30 September 2024 for articles assessing PPI deprescribing in adult patients, focusing on the implementation rate (primary outcome) or effects on symptoms (secondary outcome). **Results**: After screening, 66 studies were included, predominantly pragmatic trials (N = 32) or randomized controlled trials (N = 25). We found a variety of interventions promoting PPI deprescription. Collaborative efforts involving multiple healthcare professionals, the use of algorithms for clinical decision-making, and patient involvement have proven to be key elements in the most effective strategies. Discontinuing therapy may not be advisable in cases of recurrent symptoms, suggesting that on-demand therapy could be a recommended approach. Deprescribing is particularly relevant for individuals with mild illnesses and symptoms, where tapering can effectively mitigate the rebound symptoms often associated with abrupt discontinuation. **Conclusions**: Given the current prevalence of inappropriate PPI prescribing, it is imperative to raise awareness among both physicians and patients about the importance of the deprescribing process, which should be tailored to the specific needs of each patient, considering his/her medical history, current health status, and personal preferences.

## 1. Introduction

Proton pump inhibitors (PPIs) are the most widely used drugs to treat or prevent acid-related conditions, such as gastroesophageal reflux disease (GERD), antiplatelet or nonsteroidal anti-inflammatory (NSAID) drug-induced ulcers, Zollinger-Ellison syndrome, and to eradicate Helicobacter Pylori (HP) [1,2]. GERD is a common digestive disorder, affecting more than one in five subjects worldwide, with Italy being among the countries with the highest prevalence rates [3] and exhibiting significant utilization of related pharmacological treatments. Indeed, in Italy, PPIs were within the top five drugs in terms of expenditure and consumption in 2022, with 18% prevalence of use, 76 Defined Daily Doses (DDD)/1000 inhabitants/day, and EUR 153 per patient, steadily increasing in recent years [4].

In several clinical trials, PPIs have been shown to be potent and effective, with an excellent safety profile [5]. This has contributed to their overuse across various treatment areas, including for conditions without a documented diagnosis. Additionally, the failure to regularly reassess the need for continued therapy—especially in patients treated by multiple healthcare providers—the underutilization of on-demand and step-down approaches, and, in some cases, the fear of symptoms recurrence all contribute to the persistent overuse of PPIs [6,7,8,9].

Long-term use of PPIs has been associated with an increased risk of conditions such as Clostridium difficile infection, pneumonia, chronic kidney disease, vitamin and mineral deficiencies and fractures [10], as well as cardiovascular events [11,12]. This is of particular concern, as PPI treatment without proper indication or extended without a recognized indication could expose patients to an increased risk of adverse drug reactions (ADRs).

Peptic ulcer disease treatment guidelines recommend short-term PPI use for most patients (up to 12 weeks); after this period, PPI therapy should be discontinued unless maintenance therapy is clearly indicated (for example, in patients with gastrointestinal risk factors, or with daily NSAID use) [13,14]. This process, also called ‘deprescribing’, has been defined as the withdrawal of an inappropriate medication, supervised by a health care professional, with the goal of managing polypharmacy and improving outcomes [15].

Although the effectiveness of deprescribing interventions has been well-established [16] and multiple recommendations have been issued by scientific societies, deprescribing is poorly implemented in clinical practice [17]. This is due in part to the variability in approaches and tools used, as well as to the lack of standardized guidelines for clinicians. As a result, there is a significant gap between the evidence supporting deprescribing and its real-world application. To better understand the reasons behind this gap and to identify the most suitable approaches for different settings, we conducted a systematic review of the existing literature to provide a comprehensive overview of deprescribing interventions of PPI therapy implemented in different settings and by different healthcare professionals.

## 2. Materials and Methods

A systematic review of the literature was conducted according to the PRISMA (Preferred Reporting Items for Systematic reviews and Meta-Analyses) statement guidelines [18]. The protocol was submitted to the PROSPERO website (ID 500774).

A systematic search of the literature was performed in the PubMed, Embase, and Web of Science databases for articles published from 1 January 1989 (the first year of PPI commercialization) to 30 September 2024. In addition to the electronic searches, the references of all included articles were crosschecked. The search strategy combined headings and keywords identified according to the PICOS (Population, Intervention, Comparison, Outcome, Study design) Model [19,20], including the following: studies on adult patients receiving any PPI deprescribing approaches; evaluating any type of PPI deprescribing intervention, addressing different healthcare professionals (i.e., physicians, clinicians, pharmacists, nurses) or patients, in different settings (i.e., nursing homes, hospitals, pharmacies); compared with no intervention or standard care; reporting implementation rate (primary outcome) or effects on symptoms (secondary outcome); designed as clinical trials and observational studies. The Boolean operators AND/OR were employed. An example of this searching strategy is reported in the Supplementary Materials section.

All original, peer-reviewed articles from within the time frame responding to the PICOS model were included in the research. Conference proceedings, rationale or design, letters, editorials, commentaries, reviews, consensus, and study protocols were excluded. Papers written in languages other than English and Italian were excluded.

According to the PICOS (Population, Intervention, Comparison, Outcome, Study design) Model, our review included studies on adult patients receiving any PPI deprescribing approaches and reporting the efficacy of the intervention vs. standard care, in terms of implementation rate (percentage of actually deprescribed patients; primary outcome) or effects on symptoms (secondary outcome). No limitations were set with regards to the setting, type of deprescribing intervention, or study design.

The study selection (title/abstract screening and full-text screening) was performed by four reviewers independently. Any disagreements between reviewers were discussed until a consensus was reached. For each article, the following characteristics were extracted: first author, year of publication, country, design, number and type of subjects involved, and main results. Studies were classified as randomized controlled trials (RCTs, explanatory trials with selected subjects randomized to one or more intervention groups and a control group), pragmatic trials (intervention studies implemented in real-world practice, often without a control group), or pre–post studies (when the effectiveness of the intervention was obtained by comparing the same measurement before and after the intervention).

The methodological quality of studies included in the primary outcome evaluation was assessed using the National Institutes of Health (NIH) Study Quality Assessment Tools [21], using a specific set of NIH-tailored quality assessment tools according to the study design. The quality of observational studies was assessed according to the NIH Quality Assessment Tool for Observational Cohort and Cross-Sectional Studies, while experimental studies were evaluated using the NIH Quality Assessment of Controlled Intervention Studies tool. The forms consist of 19 and 14 questions, respectively, based on the key concepts for assessing the internal validity of a study. Each assessment question was rated with “yes”, “no”, cannot determine (CD), not applicable (NA), or not reported (NR). The final quality rating of each study was calculated based on the percentage of valid answers to the valid questions, resulting in good (66–100%), fair (33–65%), or poor (1–33%) quality. The instrument was applied independently by four reviewers. Divergent opinions were discussed among authors until a consensus was reached.

To characterize and evaluate the heterogeneity of deprescribing interventions, the results were described not only based on the pre-specified outcomes, but also by assessing the type of intervention implemented, the mode of delivery (whether directly to the patient or through a healthcare professional), and the healthcare professionals involved, with particular emphasis on multidimensional interventions.

## 3. Results

**Figure 1:**
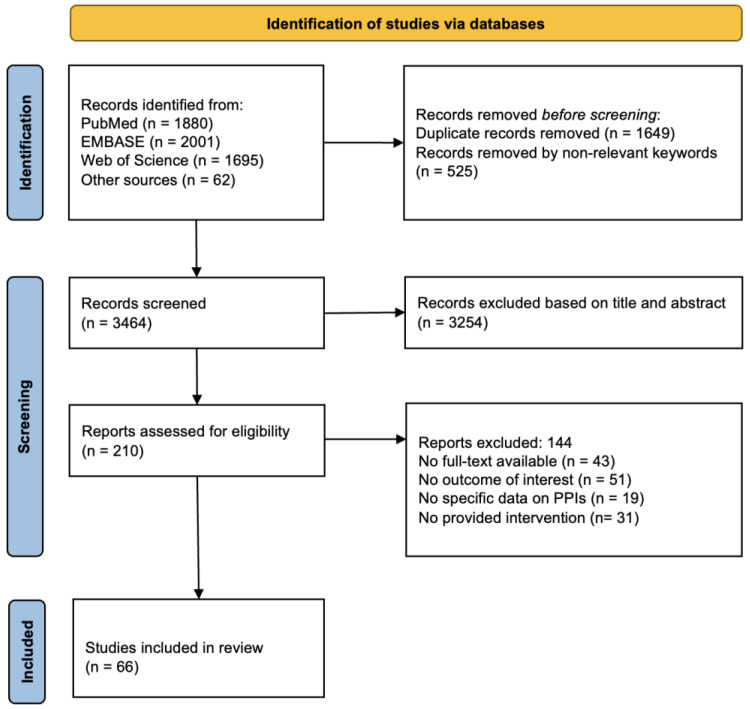
PRISMA diagram of the review’s systematic search results.

The results of the search strategy are depicted in Figure 1. After screening, 66 eligible studies were identified (Table 1). Overall, 29 studies were from Europe, 26 were from the U.S./Canada, 8 were from Asia, and 3 were from Australia. The majority of the studies were pragmatic trials (N = 32) or randomized controlled trials (N = 25). The primary outcome was reported by 28 studies, while the secondary outcome was reported by 29 studies; 9 studies reported both primary and secondary outcomes. Depending on the aim of the study, the patients involved were mostly PPI users or users with a specific disease (in most cases, GERD). Quality was rated as ‘good’ for 53 out of 66 studies.

### 3.1. Results on Primary Outcome

To evaluate the implementation rate, we selected 37 studies in which deprescribing interventions consisted of therapy evaluations and/or recommendations for changes provided to the physician by another healthcare professional (often a pharmacist), with the final decision left to the physician, or interventions involving the physician making a recommendation to the patient, who was then free to decide whether or not to follow it. Heigh RCTs reported the primary outcome, with the implementation rates ranging from 24% up to 67%. The lowest values were reported for interventions provided directly to the patients—through oral recommendations [36] or e-mails [30]—or for an intervention addressing inappropriate prescribing (not specifically with PPIs) during hospitalization in older patients with multimorbidity [81] (good quality for all studies). The highest value was reported by Potter et al. [59] (good quality) in a study where a unique pharmacist-managed PPI tapering schedule was developed and implemented in 22 patients to deprescribe unnecessary PPI therapy. In the other 29 studies, the implementation rates ranged from 38% [48] (good quality) to over 90% [42,61] (good quality).

The included studies showed a wide variety of intervention types. In a minority of the studies, the interventions directly involved the patient, with advice and recommendations provided by the pharmacist or the doctor [30,36,51,66,69,73,82,86]. In most cases, the intervention recipients were the physicians, with educational interventions targeting General Practitioners (GPs) [46,50,68,72], or general recommendations for reassessing PPI treatment and determining whether continued administration was actually necessary [26,40]. Sometimes, this re-evaluation was entrusted to a clinical pharmacist [6,40,43,48,56,61,65,67,71,74,75,87]. In other cases, physicians were provided with a support tool, such as deprescribing guidelines [57,58,59,64] or software/algorithms [42,45,53,58,60,70,76,80,84,85]. For example, in the study by Garfinkel and Mangin [42], the Good Palliative–Geriatric Practice algorithm was applied to a cohort of 70 community-dwelling older patients to recommend drug discontinuation. It was an implicit tool, not specific for PPIs, designed to guide clinicians through determining the appropriateness of a medication and provide advice on whether to stop, reduce the dose, continue, or switch to an alternative. The algorithm was a decision tree based on the rationale for recommendations, the level of evidence for a positive benefit-to-risk ratio, and its possible impact on longevity and quality of life. In the study by Curtain et al. [43], the tool was a software-generating computerized decision support prompt. The prompt was programmed to appear to pharmacists every time one of the specified products was chosen during the dispensing process. It advised pharmacists to discuss with eligible patients the possibility of reducing their medication to a lower dosage, on consultation with their GP. The prompt contained links to printable leaflets targeted at GPs and patients.

Some pilot studies implemented more elaborate methodologies/approaches in specific settings; in the study by Nallapeta et al. [70], a multidisciplinary quality improvement team used the Plan-Do-Study-Act Model of health care improvement and performed a root cause analysis to identify the barriers to inappropriate use of PPIs. A customized electronic health record template was created to design a workflow for nursing staff to remind providers to assess PPI use. All these instruments involve discussion with the patient in order to reach a mutually agreed-upon final decision [42,43,51,65,66,69,81,84]. In the study by Coyle et al. [66], adult PPI-treated patients were invited to a 20-min dyspepsia clinic appointment with a trained nurse adviser. An action plan to reduce and/or stop their PPI usage was discussed. In the study by Krol et al. [30], a simple information leaflet was sent directly to patients, containing information about the updated recommendations made to GPs regarding the clinical management of dyspepsia and emphasizing the importance of reducing the inappropriate use of PPIs. Suggestions were made to reduce or stop using PPIs and advice was given on when to seek help from their GP.

### 3.2. Results on Secondary Outcome

A critical point in the deprescription of PPIs is the possibility of gastric symptomatology rebound. For this reason, several studies have evaluated this issue, comparing different ways of discontinuing therapy, such as reducing the dosage, switching to as-needed use, or discontinuing it permanently.

The evaluation of secondary outcomes encompassed 38 studies, of which almost 90% (33/38) were of good quality. Six pragmatic trials applied deprescribing protocols with abrupt PPI discontinuation [61,62,75,76,77,79]; seven pragmatic trials tested a step-down approach [23,24,25,45,87], on-demand usage [27,31], or both [44]. Two studies compared abrupt vs. gradual withdrawal [36,82]. In all studies, the majority of patients (51–88%) did not report any recurring symptoms of heartburn or acid regurgitation during the observation period. Three RCTs compared on discontinuation vs. daily therapy [41,52,55], reporting a rate of recurrence symptoms in the discontinuation arm of 20–68%. Three RCTs compared discontinuation vs. on-demand therapy [22,28,29], reporting a rate of recurrence of symptoms in the discontinuation arm of 20–44%. The comparison between on-demand PPIs vs. daily treatment was investigated in 12 RCTs, with mixed results: 6 RCTs [33,34,35,47,54,63] reported that the symptom relief rate was non-significantly different between continuous and on-demand treatment groups, while in the other 6 RCTs [32,37,38,39,49,83], the GERD symptom and health-related quality of life scores were significantly higher in the continuous treatment group.

These findings were confirmed by RCTs. In the trial by Krol et al. [30], an information leaflet sent to chronic PPI users resulted in 24% of patients stopping or reducing their treatment, compared to 7% in the control group of standard care, without changes in dyspepsia symptom severity and quality of life after 12 weeks. Similar results were obtained in the RCT by Tarabay et al. [80], where the reported breakthrough symptoms in participants who stepped down or off PPI treatment decreased over time. Björnsson et al. [36] evaluated whether long-term PPI users (mainly for GERD) could discontinue the medication without developing symptoms. Overall, 27% of patients did not use PPIs during the year following discontinuation, without significant differences observed between patients randomized to tapering and those without tapering. Despite gastrointestinal symptom rating scales and Glasgow dyspepsia scores being similar at baseline in those who resumed PPIs compared to non-resumers, the discontinuation of PPIs was associated with a significant positive impact on quality of life.

Focusing on endoscopic outcomes, the results suggest that the success of deprescribing approaches is closely linked to the severity of the underlying condition. Sjöstedt et al. [32] compared on-demand esomeprazole to once-daily therapy for maintaining endoscopic remission in patients with healed erosive esophagitis over a 6-month period, showing that daily therapy was more effective, particularly in more severe cases of esophagitis. In Abu Farsakh [24], follow-up endoscopy in patients who underwent step-down PPI therapy showed improvement in esophagitis grade for those with controlled symptoms. Similarly, Cho et al. [63] found that 12 weeks of on-demand esomeprazole (40 mg) was not inferior to continuous therapy for symptom relief. However, they noted that the optimal doses and durations for both on-demand and continuous PPI therapy remain unclear. Some authors suggested that discontinuation of these drugs should be restricted to PPI users without robust indication for the drug, particularly if they do not have symptoms of GERD. This evidence was confirmed in the RCT by Reimer et Bytzer [41], in which 68% of patients experienced symptom recurrence after PPI discontinuation, leading authors to conclude that discontinuation of long-term PPI therapy is possible in a minority of primary care patients.

## 4. Discussion

PPIs are acknowledged to be among the most widely used medications; however, they are also linked to inappropriate use, either due to being prescribed without a clear indication or being extended beyond the necessary duration [88,89]. For these reasons, PPI deprescribing has become central in the clinical literature, especially in the last decade. Drawing up deprescription guidelines is beyond the scope of this review. Several scientific societies and expert groups have proposed recommendations to make doctors aware of the importance of discontinuing treatment when it is not necessary [90,91,92]. However, there is no clear guidance on how to promote the practice of deprescribing. This is primarily due to the fact that different settings may have access to varying data, tools, and budgets, and interventions are tailored accordingly. This leads to the substantial heterogeneity observed.

Our systematic review provides up-to-date data regarding the approaches of PPI deprescribing. Despite the high methodological heterogeneity of the studies included, some conclusions can be drawn.

We found a high variability in the types of PPI deprescription approaches. Obviously, as with the initial prescription, the process of deprescribing should also be informed by a comprehensive evaluation of the patient’s condition and based in the end on the clinician’s judgment. Therefore, it is not possible to recommend a universal strategy [76]. However, evidence shows that it is possible to facilitate physicians’ choices. Tools such as deprescription guidance algorithms have the advantages of being easy to use, supporting and guiding the clinician’s decision, and involving patients [51], as algorithms usually encompass a shared decision process. The limited availability of guidelines for deprescribing is one of the main system-related barriers reported by physicians [93,94,95], and in recent years this gap has been partly filled by the publication of evidence-based guidelines and recommendations [90,91]. However, it is crucial for doctors to be trained to routinely implement this approach, since prescribers’ attitudes and/or experience are reported as another main system-related barrier. Notably, this practice takes time [42], and time constraints are considered as major impediments facing the implementation of deprescribing strategies [95].

Nevertheless, many interventions, differing in methodology and setting, proved to increase deprescription rates and reduce inappropriate prescriptions. This evidence suggests that there are many possible ways to improve PPI prescribing practices and that the most appropriate approach is probably to design interventions that are better suited to a specific organizational and cultural context. For example, in settings where collaboration between pharmacists and doctors is high, pharmacist-supported deprescription can be highly effective [96,97]. As reported by Del-Pino et al. [98], interventions led by interprofessional healthcare teams (such as physicians, pharmacists, nurses, or others) had a higher deprescribing success rate compared to single-professional led strategies, thus highlighting the potential for a collaborative environment. Otherwise, in contexts where the digitalization of medical data is advanced, the application of decision support software based on such data would be strategic [99]. In such cases, although the application of explicit criteria (such as Beers or STOPP) [100] may guide the identification of patients, it is essential that a thorough review of treatment is then conducted by the relevant healthcare personnel. The choice of the most suitable strategy requires health institutions and decision-makers to have a thorough understanding of the characteristics of each specific context and the attitudes of the professionals working in it.

Our review also showed that, despite the majority of effectively deprescribed patients found, there was a proportion of patients in which deprescribing failed. A potential explanation could be related to physiological changes triggered by the PPI treatment itself, which are set off once therapy is withdrawn. A study on healthy volunteers demonstrated that treatment with a PPI for 8 weeks induces acid-related symptoms like heartburn, acid regurgitation, and dyspepsia once treatment is withdrawn [101]. This evidence justifies the results obtained with gradual withdrawal approaches, such as tapering or switching to on-demand therapy [34]. However, differences in the rates of therapy resumption after discontinuation are mainly based on the criteria for patient selection. High percentages of deprescribed patients and lower rates of symptom relapse occurred when subjects were selected upon inappropriateness of treatment, or when deprescribing was focused on patients with uncomplicated GERD, non-erosive esophagitis, or mild recurrent symptoms, resulting in greater benefits from deprescribing approaches [38]. Conversely, in subjects with clinically proven gastric disease (e.g., erosive GERD, which shows the highest symptom relapse rate) continuous treatment has often proven more effective in controlling symptoms, and in fact is preferred by affected patients [36]. Nocon et al. [102] found that the initial diagnosis of erosive GERD was associated with a higher probability of continuous treatment compared to on-demand treatment. Other factors associated with deprescribing failure included longer PPI duration [25] and symptom severity [36,44]. In the study by Wu et al. [44], irritable bowel syndrome, in addition to daily reflux symptom and concomitant dyspepsia, were independent predictors for deprescribing failure. Overall, these data seem to suggest that in some situations, complete withdrawal of PPI therapy may not be appropriate. In the study by Lindt et al. [22], approximately 50% of patients with heartburn who did not have esophagitis needed acid inhibitory therapy in addition to antiacid medication to preserve a normal quality of life. In these patients, on-demand therapy could be an effective treatment strategy [28].

Notably, deprescription failure in managing gastric symptoms may also be linked with poor patient adherence to lifestyle recommendations. It is important that the discontinuation of PPI therapy is accompanied by an educational intervention with the patient [90,103]. In this respect, patient communication plays a crucial role in the deprescribing process, not only to provide accurate guidance on managing any rebound symptoms, but also to ensure that the clinical decision is shared and that patients can effectively have a proactive role in managing their own health. For instance, on-demand therapy has been found to be associated with significant patient satisfaction [48]. Several studies [23,31,75] have reported high level of patient acceptance for deprescribing. In the study by Ayoub et al. [75], collaboration between pharmacists and primary care physicians enabled the development of patient-specific PPI tapering plans that were convenient and well-received by patients.

To our knowledge, this is the first study to have systematically searched and discussed the available evidence on deprescribing approaches for PPI therapy. The choice to include different types of studies (both RCTs and pragmatic trials) allowed us to gather evidence on different types of interventions tested in daily practice and to collect information on the extent to which they were applied in clinical routine. Our review provides comprehensive and updated information to better understand the complexity of deprescribing practices and to guide potential interventions to improve this process. The findings are limited by the high variability of methodological approaches, patient characteristics, and study design. The fact that most of the studies were pragmatic trials makes it challenging to obtain a robust and reliable pooled quantitative estimate, as well as to compare effectiveness across various studies. However, the variability of interventions has the merit of offering a multiplicity of instruments that can be selected from and adapted to different contexts.

## 5. Conclusions

Our review showed that several different interventions can be implemented to foster PPI deprescription. The interventions encompassing collaboration between multiple healthcare professionals, exploiting algorithms to guide clinical decision-making, and involving patients have been shown to be effective and valued by stakeholders. Results also seem to suggest that therapy should not be discontinued in some cases with recurrent symptoms, but that these patients typically benefit from on-demand therapy. Deprescribing is mostly applicable to individuals with mild illnesses and symptoms, and symptom rebounds that occur with the abrupt discontinuation of therapy can be effectively avoided with tapering. In conclusion, deprescribing is a complex process that should be tailored to each patient’s needs. It is important to consider the patient’s specific medical history, current health status, and preferences while striving to optimize their medication regimen and overall well-being.

## Figures and Tables

**Table 1:** Summary of studies (n = 66) evaluating deprescribing interventions on PPI therapy.

First Author, Year	Country	Study Design	Patients	Setting	Intervention (I) or Control (C)	Outcome	Results	Quality Rating
Lind T, 1999 [22]	Denmark and Sweden	Randomized controlled trial	424 patients with GERD	Healthcare sites	(I) PPI discontinuation (placebo); (C) on-demand PPI therapy	SO	After 6 months the remission rates were 83% with omeprazole 20 mg, 69% with omeprazole 10 mg, and 56% with placebo (*p* < 0.01 for all intergroup differences).	Good
Inadomi JM, 2001 [23]	Mexico	Pragmatic trial	71 patients with GERD	General practice	Step-down approach	SO	After 1 year of FU, 58% were asymptomatic off PPI therapy.	Good
Abu Farsakh N, 2003 [24]	Jordan	Pragmatic trial	142 patients with heartburn	University Health Center	Step-down approach	SO	After 36–54 months of FU, 51% of patients were symptomatically controlled without PPI: 28% had no treatment or received occasional antacids or H2-blockers, and 23% required frequent H2-blockers.	Good
Inadomi JM, 2003 [25]	US	Pragmatic trial	117 patients with heartburn or acid regurgitation	Medical center and outpatient facilities	Step-down approach	SO	During the 6 months after step-down to single-dose PPI, 79.5% did not report recurrent symptoms of heartburn or acid regurgitation.	Good
Pohland CJ, 2003 [26]	US	Pragmatic trial	248 patients with lansoprazole prescriptions	Veteran Health Care	Recommended step-down approach + patient education	PO	Interventions for step-down therapy were recommended for 120 patients. 46% of recommendations were implemented.	Good
Ponce J, 2004 [27]	Spain	Pragmatic trial	51 patients with GERD	Clinical practice	On-demand therapy	SO	After 6 months of FU, symptom control was achieved in over 85% of the patients	Good
Scholten T, 2004 [28]	Austria, The Netherlands, Germany	Randomized controlled trial	634 patients with endoscopically confirmed GERD	Healthcare sites	(I) PPI discontinuation (placebo); (C) on-demand PPI therapy	SO	The perceived average daily symptom load and the number of antacid tablets taken were significantly higher in the placebo than in both pantoprazole groups (*p* < 0.0001).	Good
Bytzer P, 2004 [29]	Multicenter	Randomized controlled trial	432 patients with NERD	Healthcare sites	(I) PPI discontinuation (placebo); (C) on-demand PPI therapy	SO	During on-demand treatment, rates of discontinuation because of inadequate heartburn control were 20% for placebo vs. 6% for rabeprazole (*p* < 0.00001).	Good
Krol N, 2004 [30]	The Netherlands	Randomized controlled trial	113 chronic PPI users	General medical practice	(I) Patient-directed intervention (direct mail); (C) standard care	PO/SO	24% of intervention group patients stopped or reduced their use of proton pump inhibitors, compared with 7% of control group patients. Dyspepsia symptom severity and quality of life did not change.	Good
Scholten T, 2005 [31]	Germany	Pragmatic trial	234 patients with GERD	General practice	On-demand therapy	SO	After 6 months of FU, 82% of patients stated that improvements in their symptoms were maintained.	Good
Sjöstedt S, 2005 [32]	Sweden	Randomized controlled trial	477 endoscopically healed patients from erosive reflux esophagitis	Hospital clinics	(I) on demand PPI therapy; (C) daily PPI therapy	SO	At 6 months, 81% of patients with daily treatment were in remission, compared with only 58% who took on-demand treatment (*p* < 0.0001).	Good
Janssen W, 2005 [33]	Germany, France, Switzerland, Hungary	Randomized controlled trial	558 endoscopically proven GERD patients	Healthcare centres	(I) on demand PPI therapy; (C) daily PPI therapy	SO	After 24 weeks of treatment, on-demand treatment was noninferior to continuous treatment.	Good
Bour B, 2005 [34]	France	Randomized controlled trial	152 patients with frequent symptomatic relapses of mild to moderate GERD	Hospital centres	(I) on demand PPI therapy; (C) daily PPI therapy	SO	At month 6, the symptom relief rate was non-significantly different between continuous and on-demand treatment groups.	Fair
Cibor D, 2006 [35]	Poland	Randomized trial	60 patients with NERD	University hospital	(I1) on-demand; (I2) daily treatment; (I3) intermittent therapy (four-week courses during a relapse)	SO	After 12 months of FU, on-demand and daily treatment models of maintenance therapy showed a similar high efficacy in controlling symptoms, whereas intermittent therapy was significantly less effective.	Good
Björnsson E, 2006 [36]	Sweden	Randomized controlled trial	96 patients on long-term PPIs, without a history of peptic ulcer or esophagitis	Pharmacies	(I) step-down then discontinuation; (C) daily PPI therapy then discontinuation	PO/SO	A total of 27% did not use PPIs during the year after discontinuation, without significant differences in gastric symptoms between the two arms.	Good
Morgan DG, 2007 [37]	Canada	Randomized controlled trial	268 patients with GERD	Healthcare sites	(I) on demand PPI therapy; (C) daily PPI therapy	SO	Results based on symptom assessments favor continuous therapy.	Fair
Tepes B, 2009 [38]	Slovenia	Randomized controlled trial	196 patients with GERD	Outpatient clinics and hospital centers	(I) on demand PPI therapy; (C) daily PPI therapy	SO	In patients without esophagitis or with esophagitis Los Angeles grade A at baseline, a statistically significantly lower relapse rate was observed with daily treatment. In patients with esophagitis Los Angeles grade B, no differences in the relapse rate were found.	Fair
van der Velden AW, 2009 [39]	The Netherlands	Randomized controlled trial	288 patients with GERD	General medical practice	(I) on demand PPI therapy; (C) daily PPI therapy	SO	In the daily pantoprazole arm, 8% were discontinued for inadequate relief vs. 24% in the placebo arm.	Good
Ramser KL, 2009 [40]	US	Pragmatic trial	129 patients with pantoprazole prescription	Internal medicine clinic	Recommended step-down approach or discontinuation	PO	At the 8-month FU, 11% of patients with suggested discontinuation and 16% of patients with suggested step-down treatment had resumed PPI therapy.	Fair
Reimer C, 2010 [41]	Denmark	Randomized controlled trial	78 PPI users	Hospital	(I) PPI discontinuation (placebo); (C) daily PPI therapy	SO	A total of 11 of 78 (14%) patients discontinued the therapy successfully. 53 of 78 patients (68%) experienced symptom recurrence.	Good
Garfinkel D, 2010 [42]	Israel	Pragmatic trial	18 community-dwelling older patients	Community day care center	Algorithm for physicians to recommend drug discontinuations	PO	90% of 10 recommended discontinuations for omeprazole therapy were performed.	Good
Curtain C, 2010 [43]	Australia	Randomized controlled trial	High-dose PPI prescribed patients	Community pharmacy	(I) Computerized clinical decision support system; (C) standard care	PO	During the 12-week trial, 1.67 step-down interventions per 100 high-dose targeted PPI prescriptions were identified in the intervention group, compared to 0.17 in the control group. A total of 63% had reviewed their medication therapy in consultation with their GP.	Good
Wu JCY, 2011 [44]	Hong Kong	Pragmatic trial	265 patients with GERD	Gastroenterology clinic	Step-down on-demand therapy	SO	During 26 weeks of FU, symptom control was maintained in 71% of the patients.	Good
Fass R, 2012 [45]	Multicenter	Pragmatic trial	142 patients with GERD	Healthcare sites	Step-down from twice-daily to once-daily PPI treatment	SO	After step-down, heartburn remained well controlled in 88% of patients.	Good
Hamzat H, 2012 [46]	Scotland	Pre-post intervention study	164 patients admitted to hospital	Hospital geriatric unit	Physician education	PO	Frequency of interventions in patients with inappropriate PPI prescribing (stopping PPI or reducing the daily dose) increased from 9% in the pre-education phase to 46% in the post-education phase.	Good
Nagahara A, 2013 [47]	Japan	Randomized controlled trial	117 patients with GERD	General medical practice	(I) on demand PPI therapy; (C) daily PPI therapy	SO	Symptom relief in cont/on-demand therapy groups were 57.6%/48.3% at baseline and 66.7%/74.0% at 24 weeks (n.s.). On-demand therapy seemed sufficient as a maintenance therapy in NERD patients. Regarding RE, continuous therapy would be recommended in terms of reduced symptoms and maintaining mucosal healing.	Good
Bundeff AW, 2013 [48]	US	Pragmatic trial	117 patients with PPI prescriptions	Multispecialty medical groups, primary care	Recommendations for physicians by clinical pharmacists	PO	At 5 months, only 37.6% of patients began the PPI taper protocol as advised by their primary care providers.	Good
Nagahara A, 2015 [49]	Japan	Randomized controlled trial	82 endoscopically proven GERD patients	Hospital	(I) on demand PPI therapy; (C) daily PPI therapy	SO	At 4, 5, 6, and 17 weeks, the continuous arm achieved more significant symptom-relief than the on-demand arm.	Fair
McDonald EG, 2015 [50]	Canada	Pre-post intervention study	640 patients adimitted to hospital	University teaching hospital	Physician education	PO	The proportion of PPIs discontinued at hospital discharge increased from 7.7% per month in the 6 months prior to intervention to 18.5% per month postintervention (*p* = 0.03).	Good
Reeve E, 2015 [51]	Australia	Pragmatic trial	57 PPI users	Hospital outpatient clinics	Patient-centered deprescribing process proposed to physicians	PO	Out of 8 patients suitable for withdrawal, 6 consented. All 6 successfully ceased or reduced their PPI use, and this was sustained at 6 months postintervention.	Fair
Zwisler JE, 2015 [52]	Denmark	Randomized controlled trial	165 patients treated with antisecretory drugs on a long-term basis	General medical practice	(I) PPI discontinuation (placebo); (C) continuous PPI therapy	SO	At 12 months, 73% of patients in the placebo group had discontinued due to the need to change back to the usual antisecretory medication, compared with 21% of the esomeprazole group (*p* < 0.001).	Good
Clyne B, 2015 [53]	Ireland	Randomized controlled trial	190 patients with potentially inappropriate prescribing	Primary care	(I) Multi-drug intervention: academic detailing; review of medicines with web-based treatment algorithms that provide recommended alternative-treatment options; and tailored patient information leaflets; (C) standard care	PO	Inappropriate PPI utilization in the intervention group significantly decreased from 53.5 to 23.2%; it did not change in control group (from 67.7 to 47.4%).	Good
Bayerdörffer E, 2016 [54]	Austria, France, Germany, South Africa, Spain	Randomized controlled trial	598 patients with NERD	General medical practice	(I) on demand PPI therapy; (C) daily PPI therapy	SO	Discontinuation due to unsatisfactory treatment was 6.3% for on-demand and 9.8% for continuous treatment. In total, 82.1 and 86.2% of patients taking on-demand and continuous therapy, respectively, were satisfied with the treatment of heartburn and regurgitation symptoms (*p* = NS).	Good
Helgadóttir H, 2016 [55]	Iceland	Randomized controlled trial	100 patients with endoscopically verified erosive esophagitis	University Hospital	(I) step-down PPI therapy; (C) daily PPI therapy	SO	The chance of failing to maintain symptom control was 24% in the reduction group and 13% in the control group (13%) (*p* = 0.2).	Good
Michal J, 2016 [56]	US	Pre-post intervention study	189 patients receiving PPIs while hospitalized	Community Hospital	Pharmacist-driven protocol for physicians to decrease PPI use	PO	PPIs were discontinued in 66.0% of postintervention group patients compared to 41.1% of the preintervention group (*p* = 0.001). 73% of recommendations were accepted.	Fair
Thompson W, 2016 [57]	Canada	Pragmatic trial	205 PPI prescriptions	Long-term care home	PPI deprescribing guideline for physicians	PO	Intervention was associated with a decrease in PPI prescribing; however, the reduction in use mainly occurred within 6 months after the start of the intervention and use gradually climbed back up, resulting in no significant difference in use by the end of the study period.	Fair
Walsh K, 2016 [58]	Canada	Pre-post intervention study	46 PPI users	Academic primary care clinic	Remind for physicians to reassess therapy via electronic medical record (EMR) messaging and a PPI Deprescribing Tool	PO	The number of patients taking a PPI without an indication decreased from 12 at baseline to 4 at the end of the project.	Good
Potter K, 2016 [59]	Australia	Randomized controlled trial	19 patients eligible for PPI deprescribing	Residential aged care facilities	(I) Multi-drug deprescribing algorithm; (C) Standard care	PO	Of 15 withdrawals attempted, 67% were successfully discontinued.	Good
McIntyre C, 2017 [60]	Canada	Pragmatic trial	23 hemodialysis patients	Tertiary-care outpatient hemodialysis unit	Deprescribing tool	PO	67% of PPIs were completely deprescribed.	Good
Lee C, 2017 [61]	Canada	Pragmatic trial	28 taking a PPI for longer than 6 months	Residential care facility	Prescription audit and recommendations to discontinue PPI without tapering by pharmacist	PO/SO	The recommendation was accepted by 96%. Eight weeks after discontinuing PPI therapy, 70% were still symptom-free and did not require PPI re-initiation.	Good
Wahking RA, 2018 [62]	US	Pragmatic trial	220 hospitalized patients not meeting criteria for inpatient PPI continuation	Internal medicine service	Inpatient PPI stewardship program	SO	95.9% tolerated inpatient PPI discontinuation, with 83.4% not requiring any “as needed” acid suppressive therapy during hospitalization. Three months after discharge, discontinuation and dose de-escalation were tolerated in 57.1% and 81.8% of patients, respectively.	Good
Cho JH, 2018 [63]	South Corea	Randomized controlled trial	80 patients with endoscopically confirmed GERD	University hospital	(I) on demand PPI therapy; (C) daily PPI therapy	SO	Intensities and frequencies of heartburn and regurgitation responded well to maintenance treatment in patients in the on-demand and continuous groups.	Good
Avraham O, 2018 [64]	US	Pragmatic trial	10 PPI elderly users	Geriatric residence	Stepwise taper protocol	PO	Physicians accepted >95% of interventions, and 90% of patients achieved cessation at 12 weeks.	Fair
Coffey CP, 2019 [65]	US	Pragmatic trial	185 patients ≥65 years with an active PPI prescription for longer than eight weeks	Academic medical center	Tapering individualized plan to patients by pharmacist	PO	The provider approval rate of the pharmacist-recommended intervention was 86%, and 103 patients initiated the taper. Of these, 81.6% were successfully weaned off their PPI.	Good
Coyle C, 2019 [66]	UK	Pragmatic trial	4691 adult patients, treated with PPIs for ≥2 consecutive months	Regional primary care organisations	Clinic appointment with a trained nurse adviser to share an action plan to reduce and/or stop PPI usage	PO	After 12 months, 75.1% of 6249 eligible patients stepped down or off PPIs.	Good
Tandun R, 2019 [67]	Canada	Pragmatic trial	58 PPI users	Long-term care facilities	Weekly reviews by pharmacist with deprescribing recommendations.	PO	Four months post-intervention, 80.0% of the 30 residents who were initiated on PPI deprescribing orders had completed them successfully by the end of the study period.	Good
Walker JM, 2019 [68]	US	Pre-post intervention study	263 PPI users	Hospital and veteran medical center	Educational intervention on gastroenterology trainees	PO	During the 8-week intervention phase, a successful intervention was performed in 42 cases, decreasing inappropriate PPI use from 49% to 33%.	Good
Boster J, 2020 [69]	US	Pragmatic trial	322 PPI users	Internal medicine clinics at multi-center military hospital system	Discussion about the risks and benefits of ongoing PPI use between physicians and patients	PO	After the six-month intervention period, 44% (96/217) of patients without a guideline recommended indication for PPI use were successfully weaned to a reduced dose or were no longer using a PPI.	Good
Nallapeta N, 2020 [70]	US	Pragmatic trial	201 PPI users	Internal medicine clinic	Customized electronic health record templates and education to providers and patients	PO	The average rate of PPI discontinuation was 51.1% (92/180), resulting in 30% inappropriate chronic PPI use from a baseline of 80% within 12 months.	Good
Odenthal DO, 2020 [71]	US	Pragmatic trial	126 patients taking a long-term PPI without a clear indication	Family medicine clinic	Pharmacist-managed PPI tapering schedule	PO	Of the 22 patients who initiated PPI deprescribing, 19 (86%) successfully discontinued their PPI completely.	Fair
Lai A, 2021 [72]	US	Pragmatic trial	187 patients prescribed inappropriate PPIs	Family medicine residency practice	Educational intervention for physicians, with frequent reminders	PO	At the 4-month FU, 100 patients inappropriately prescribed remained on PPIs (46.6% success rate).	Good
Hendricks E, 2021 [73]	US	Randomized trial	38 patients with GERD	Internal medicine and geriatric outpatient clinics	(I1) discontinuing abruptly; (I2) tapering	PO	When we combined both groups, 58% [19] were able to discontinue PPI at 12 months.	Fair
Li Wong S, 2021 [74]	Malaysia	Pragmatic controlled trial	568 PPI users	Health clinics	(I) Pharmacist with deprescribing recommendations for physicians based on patients’ questionnaire; (C) Standard care	PO	Inappropriate PPI utilization in the intervention group significantly decreased from 79.9 to 30.4%; it did not change in the control group (from 79.9 to 77.1%) (*p* < 0.05). In the intervention group, the pharmacist made 227 recommendations, and changes were made to 198 prescriptions, resulting in an 87.2% acceptance rate of pharmacist recommendations.	Fair
Ayoub J, 2021 [75]	US	Pragmatic trial	39 PPI users	Healthcare clinics	De-escalation according to pharmacist protocol	PO/SO	79% (15/19) had successful PPI de-escalation after 4 weeks without discomfort or symptoms which disrupted daily activities.	Good
Calvo LLJ, 2021 [76]	Spain	Pragmatic trial	371 PPI users	Gastroenterology department of University Hospital	Deprescribing algorithm for physician	PO/SO	Among 86 patients with inappropriate PPI prescriptions, 75 (87%) accepted the deprescribing process. Sixty-one (81.3%) maintained deprescription at week 4, 56 (74.7%) at week 12, and 54 (72.0%) at week 24. 11 of 21 restarted the PPI because of symptoms.	Good
Czikk D, 2022 [77]	Canada	Pragmatic trial	74 patients with end-stage kidney disease	Nephrology wards	Deprescribing tool	SO	Among 29 patients who agreed to a trial of PPI withdrawal, 14 restarted their PPI, most for gastroesophageal reflux disease. Three patients had a GI bleed, 1 fatally. Serum phosphate and serum magnesium increased.	Good
Tan CJY, 2022 [78]	Singapore	Pre-post intervention study	262 inpatients who were deprescribed PPIs	Tertiary hospital	Institutional PPI deprescribing guide for physicians	SO	There were no significant changes in incidence of peptic ulcer disease. In the retrospective chart review, a majority (62.6%) of patients remained deprescribed at 6 months.	Good
Tanaka H, 2022 [79]	Japan	Pragmatic trial	92 patients with gastroesophageal reflux disease	University hospital	Deprescribing intervention	SO	71.7% of patients showed no symptom relapse after drug withdrawal.	Good
Tarabay RB, 2022 [80]	Lebanon	Randomized controlled trial	140 adult patients with chronic PPI use	Family medicine center	(I) PPI deprescribing algorithm for physicians and patient-oriented informative and motivational leaflet; (C) standard care	PO/SO	At the 6-month follow-up, the rate of participants who stepped down or off PPI was higher in the intervention group (87.1% vs. 28.6%). In participants who stepped down or off PPI, the reported breakthrough symptoms decreased over time.	Good
Aubert CE, 2023 [81]	Switzerland, The Netherlands, Belgium, and Republic of Ireland	Randomized controlled trial	1080 hospitalised PPI users	University hospital	(I) pharmacotherapy optimization intervention; (C) standard care	PO	At discharge, 133 (24.9%) of 534 patients with PPIs at admission in the intervention group had deprescribing, compared with 92 (16.8%) of 546 patients in the control group.	Good
Barraquer Comes A, 2023 [82]	Spain	Pragmatic trial	109 PPI users during hospitalization	Hospital	Deprescription recommendations	PO/SO	Deprescription was performed in 49% of patients, with 64% undergoing abrupt withdrawal. Rebound symptoms led to treatment restart in 15% of cases, without significant differences in restart proportions between the abrupt and gradual withdrawal groups.	Good
Jung DH, 2023 [83]	Republic of Korea	Randomized controlled trial	304 patients with GERD	Hospital	(I) on demand PPI therapy; (C) daily PPI therapy	SO	Compared with the on-demand group, the GERD symptom and health-related quality of life scores significantly improved and the overall satisfaction score was significantly higher in the continuous treatment group.	Fair
Heisig J, 2023 [84]	Germany	Randomized controlled trial	1032 patients with regular PPI prescription for at least 6 months	General practice	(I) prescription-supporting software; (C) standard care	PO/SO	In 33.4% of consultations, GPs and patients agreed to discontinue PPI; in 15.8% of consultations, they decided to reduce the dose. 41.9% stayed on their course to stop taking PPIs 6 months later.	Good
Calvini G, Baiardi G, 2023 [85]	Italy	Pre-post intervention study	1120 patients discharged with PPI prescriptions	Hospital	Implementation and application of flowchart	PO	Results included a 73.8% reduction in patients on PPI therapy compared to the same period in the previous year.	Good
Fitzgerald Jones K, 2024 [86]	USA	Pragmatic controlled trial	4064 veterans with a chronic PPI	Veterans Affairs medical centers	(I) Medication-specific brochure for patients; (C) standard care	PO	The deprescribing rate was 29.4% in the intervention cohort vs. 25.4% in the control cohort.	Good
Mati E, 2024 [87]	France	Pragmatic trial	113 geriatric patients on PPI for more than 8 weeks	Geriatric hospital	Reassessment of PPI therapy via collegial consultation between physicians and pharmacists	PO/SO	Gradual discontinuation was performed in 54.6%. 80.9% of discontinuations were well-tolerated at 3 months	Good

I = Intervention; C = Control; GERD = Gastroesophageal reflux disease; NERD = Nonerosive reflux disease; PPI = Proton pump inhibitors; FU = Follow-up; US = United States; GP = General practitioner; RE = Reflux esophagitis; NS = Not significant; GI = Gastrointestinal; EMR = electronic medical record; PO = Primary outcome; SO = Secondary outcome.

## Data Availability

No original data were used for the research described in the article.

## Supplementary Materials

The following supporting information can be downloaded at: https://www.mdpi.com/article/10.3390/jcm13206283/s1, Supplementary Methods: Search strategy for PubMed.
