# Supplementary material for: Approaches to Deprescribing Proton Pump Inhibitors in Clinical Practice: A Systematic Review
Source: J Clin Med. 2024 Oct 21;13(20):6283. doi: 10.3390/jcm13206283 (PMC11508458; doi:10.3390/jcm13206283)
Supplement: Supplementary file 1 [file jcm-13-06283-s001.zip › jcm-3219208-supplementary.pdf]

Approaches to deprescribing proton pump inhibitors in clinical practice: a systematic review

Andrea Rossi*[1,2], Lara Perrella*[3], Stefano Scotti[2], Elena Olmastroni[1,2], Federica Galimberti[2], Ilaria Ardoino[4], Valentina Orlando[3], Enrica Menditto[3], Carlotta Franchi[4], Manuela Casula[1,2]

***co-first authors**

1 Epidemiology and Preventive Pharmacology Service (SEFAP), Department of Pharmacological and Biomolecular Sciences (DiSFeB), University of Milan, Milan, Italy.

2 IRCCS MultiMedica, Sesto San Giovanni (Milan), Italy.

3 CIRFF, Center of Pharmacoeconomics and Drug Utilization Research, Department of Pharmacy, University of Naples Federico II, Naples, Italy.

4 Laboratory of Pharmacoepidemiology and Human Nutrition, Department of Health Policy, Istituto di Ricerche Farmacologiche Mario Negri IRCCS, Milan, Italy.

Search strategy for PubMed

"PPI"[All Fields] OR "PPIS"[All Fields] OR ("proton pumps"[MeSH Terms] OR ("proton"[All Fields] AND "pumps"[All Fields]) OR "proton pumps"[All Fields] OR ("proton"[All Fields] AND "pump"[All Fields]) OR "proton pump"[All Fields]) OR (("proton pumps"[MeSH Terms] OR ("proton"[All Fields] AND "pumps"[All Fields]) OR "proton pumps"[All Fields] OR ("proton"[All Fields] AND "pump"[All Fields]) OR "proton pump"[All Fields]) AND "inhibit*"[All Fields]) OR ("rabeprazol"[All Fields] OR "rabeprazole"[MeSH Terms] OR "rabeprazole"[All Fields]) OR ("pantoprazole"[MeSH Terms] OR "pantoprazole"[All Fields] OR "pantoprazol"[All Fields]) OR ("lansoprazole"[MeSH Terms] OR "lansoprazole"[All Fields] OR "lansoprazol"[All Fields]) OR ("esomeprazole"[MeSH Terms] OR "esomeprazole"[All Fields] OR "esomeprazol"[All Fields]) OR ("omeprazole"[MeSH Terms] OR "omeprazole"[All Fields] OR "esomeprazole"[MeSH Terms] OR "esomeprazole"[All Fields] OR "omeprazol"[All Fields]) OR ("esomeprazole"[MeSH Terms] OR "esomeprazole"[All Fields] OR "esomeprazol"[All Fields] OR "nexium"[All Fields]) OR "alenia"[All Fields] OR "esoz"[All Fields] OR "esofag"[All Fields] OR "nexiam"[All Fields] OR "losec"[All Fields] OR ("omeprazole"[MeSH Terms] OR "omeprazole"[All Fields] OR "esomeprazole"[MeSH Terms] OR "esomeprazole"[All Fields] OR "omeprazol"[All Fields] OR "prilosec"[All Fields]) OR ("omeprazole sodium bicarbonate drug combination"[Supplementary Concept] OR "omeprazole sodium bicarbonate drug combination"[All Fields] OR "rapinex"[All Fields]) OR "ocid"[All Fields] OR "lomac"[All Fields] OR "omepral"[All Fields] OR "omez"[All Fields] OR ("hydrogen"[MeSH Terms] OR "hydrogen"[All Fields] OR "protium"[All Fields]) OR ("pantoprazole"[MeSH Terms] OR "pantoprazole"[All Fields] OR "pantoprazol"[All Fields] OR "protonix"[All Fields]) OR "pantozol"[All Fields] OR "pantor"[All Fields] OR "pantoloc"[All Fields] OR

"controloc"[All Fields] OR "pantecta"[All Fields] OR "somac"[All Fields] OR "zentro"[All Fields] OR ("rabeprazol"[All Fields] OR "rabeprazole"[MeSH Terms] OR "rabeprazole"[All Fields] OR "aciphex"[All Fields]) OR ("rabeprazole"[MeSH Terms] OR "rabeprazole"[All Fields] OR "dexrabeprazole"[All Fields]) OR ("rabeprazole"[MeSH Terms] OR "rabeprazole"[All Fields] OR "pariet"[All Fields]) OR "Zechin"[All Fields] OR "Rabeloc"[All Fields] OR ("lansoprazole"[MeSH Terms] OR "lansoprazole"[All Fields] OR "agopton"[All Fields]) OR ("lansoprazole"[MeSH Terms] OR "lansoprazole"[All Fields] OR "bamalite"[All Fields]) OR ("levant"[Journal] OR "levant"[All Fields]) OR ("lansoprazole"[MeSH Terms] OR "lansoprazole"[All Fields] OR "lanzor"[All Fields]) OR ("lansoprazole"[MeSH Terms] OR "lansoprazole"[All Fields] OR "monolitum"[All Fields]) OR ("lansoprazole"[MeSH Terms] OR "lansoprazole"[All Fields]) OR ("lansoprazole"[MeSH Terms] OR "lansoprazole"[All Fields] OR "ogastro"[All Fields]) OR ("lansoprazole"[MeSH Terms] OR "lansoprazole"[All Fields] OR "opiren"[All Fields]) OR ("lansoprazole"[MeSH Terms] OR "lansoprazole"[All Fields] OR "lansoprazol"[All Fields] OR "prevacid"[All Fields]) OR ("lansoprazole"[MeSH Terms] OR "lansoprazole"[All Fields] OR "prezal"[All Fields]) OR ("lansoprazole"[MeSH Terms] OR "lansoprazole"[All Fields] OR ("pro"[All Fields] AND "ulco"[All Fields]) OR "pro ulco"[All Fields]) OR ("lansoprazole"[MeSH Terms] OR "lansoprazole"[All Fields] OR ("pro"[All Fields] AND "ulco"[All Fields]) OR "pro ulco"[All Fields]) OR ("lansoprazole"[MeSH Terms] OR "lansoprazole"[All Fields] OR "promeco"[All Fields]) OR ("dexlansoprazole"[MeSH Terms] OR "dexlansoprazole"[All Fields] OR "kapidex"[All Fields]) OR ("dexlansoprazole"[MeSH Terms] OR "dexlansoprazole"[All Fields] OR "dexilant"[All Fields]) OR ("tenatoprazole"[Supplementary Concept] OR "tenatoprazole"[All Fields] OR "tenatoprazole"[All Fields]) OR "benatoprazole"[All Fields] OR ("lansoprazole"[MeSH Terms] OR "lansoprazole"[All Fields] OR "takepron"[All Fields]) OR ("lansoprazole"[MeSH Terms] OR "lansoprazole"[All Fields] OR "ulpax"[All Fields]) OR ("dexlansoprazole"[MeSH Terms] OR "dexlansoprazole"[All Fields]) OR "anadir"[All Fields] OR "antra"[All Fields] OR "cletus"[All Fields] OR "gastroloc"[All Fields] OR "lansox"[All Fields] OR "limnos"[All Fields] OR "limpidex"[All Fields] OR "losec"[All Fields] OR "lucen"[All Fields] OR "mepral"[All Fields] OR "nansen"[All Fields] OR "pantecta"[All Fields] OR ("rabeprazole"[MeSH Terms] OR "rabeprazole"[All Fields] OR "pariet"[All Fields]) OR "peptazol"[All Fields] OR "protec"[All Fields] OR "rabex"[All Fields] OR "zolium"[All Fields] OR ("lansoprazole"[MeSH Terms] OR "lansoprazole"[All Fields] OR "zoton"[All Fields]) AND (("demand"[Title/Abstract] OR "on demand"[Title/Abstract] OR "mainte*"[Title/Abstract] OR "long-term"[Title/Abstract] OR "long-term"[Title/Abstract] OR "implem*"[Title/Abstract] OR "inapprop*"[Title/Abstract] OR "approp*"[Title/Abstract] OR "withdrawal"[Title/Abstract] OR "withhold"[Title/Abstract] OR "step-down"[Title/Abstract] OR "step-down"[Title/Abstract] OR "step-up"[Title/Abstract] OR "step-up"[Title/Abstract]) AND ("on demand"[Title/Abstract] OR "demand"[Title/Abstract] OR "deprescri*"[Title/Abstract] OR "de prescr*"[Title/Abstract] OR "unprescr*"[Title/Abstract] OR "cessat*"[Title/Abstract] OR "interrupt*"[Title/Abstract] OR "stop"[Title/Abstract] OR "ceas*"[Title/Abstract] OR "suspen*"[Title/Abstract] OR "noncontin*"[Title/Abstract] OR "non contin*"[Title/Abstract] OR "de implem*"[Title/Abstract] OR "discon*"[Title/Abstract] OR "inapprop*"[Title/Abstract] OR "approp*"[Title/Abstract] OR "taper*"[Title/Abstract] OR "withdrawal"[Title/Abstract] OR "withhold"[Title/Abstract] OR "step-down"[Title/Abstract] OR "step-up"[Title/Abstract] OR "relap*"[Title/Abstract])) AND (("stud*"[Title/Abstract] AND ("observational"[Title/Abstract] OR "open label"[Title/Abstract] OR "prospective"[Title/Abstract] OR "follow-up"[Title/Abstract] OR "follow-up"[Title/Abstract] OR "cohort"[Title/Abstract] OR "longitudinal"[Title/Abstract]) OR "multicente*"[Title/Abstract]) OR "control grou*"[Title/Abstract] OR "contro*"[Title/Abstract] OR "RCT"[Title/Abstract] OR "RCTs"[Title/Abstract] OR ("tria*"[Title/Abstract] AND (("contro*"[All Fields] AND "clinical"[Title/Abstract]) OR ("rando*"[All Fields] AND "contro*"[Title/Abstract]) OR "clinical"[Title/Abstract])) OR "double blin*"[Title/Abstract] OR "double blin*"[Title/Abstract] OR "single blin*"[Title/Abstract] OR "single blin*"[Title/Abstract] OR "rando*"[Title/Abstract] OR "double-dummy"[Title/Abstract] OR "double-dummy"[Title/Abstract] OR "double-mask"[Title/Abstract] OR "double-mask"[Title/Abstract] OR "double-masked"[Title/Abstract] OR "double-masked"[Title/Abstract]) AND ("English"[Language] OR "Italian"[Language]) AND ("huma*"[Title/Abstract] OR "patien*"[Title/Abstract] OR "subject*"[Title/Abstract] OR "outpatien*"[Title/Abstract] OR "inpatien*"[Title/Abstract]) AND 1989/01/01:2023/12/31[Date - Publication] NOT (meta-analysis[Filter] OR review[Filter] OR systematicreview[Filter])
